# Optimizing and Quantifying Gold Nanospheres Based on LSPR Label-Free Biosensor for Dengue Diagnosis

**DOI:** 10.3390/polym14081592

**Published:** 2022-04-14

**Authors:** Sajid Farooq, Faiz Wali, Denise Maria Zezell, Renato E. de Araujo, Diego Rativa

**Affiliations:** 1 Center for Lasers and Applications, Instituto de Pesquisas Energeticas e Nucleares, IPEN—CNEN, Sao Paulo 05508-000, Brazil; sajiddahar@gmail.com (S.F.); zezell@usp.br (D.M.Z.); 2 Institute of Technological Innovation, University of Pernambuco, Recife 50100-000, Brazil; diego.rativa@poli.br; 3 Key Laboratory of Optoelectronic Devices and Systems of Ministry of Education and Guangdong Province, College of Physics and Optoelectronic Engineering, Shenzhen University, Shenzhen 518060, China; 4 Laboratory of Biomedical Optics and Imaging, Federal University of Pernambuco, Recife 52171-900, Brazil; renato_de_araujo@yahoo.com.br; 5 Applied Physics Program, Federal Rural University of Pernambuco, Recife 52171-900, Brazil

**Keywords:** plasmonic, nanosensor, sensitivity, figure of merit

## Abstract

The localized surface plasmon resonance (LSPR) due to light–particle interaction and its dependence on the surrounding medium have been widely manipulated for sensing applications. The sensing efficiency is governed by the refractive index-based sensitivity ($\eta_{RIS}$) and the full width half maximum (FWHM) of the LSPR spectra. Thereby, a sensor with high precision must possess both requisites: an effective $\eta_{RIS}$ and a narrow FWHM of plasmon spectrum. Moreover, complex nanostructures are used for molecular sensing applications due to their good $\eta_{RIS}$ values but without considering the wide-band nature of the LSPR spectrum, which decreases the detection limit of the plasmonic sensor. In this article, a novel, facile and label-free solution-based LSPR immunosensor was elaborated based upon LSPR features such as extinction spectrum and localized field enhancement. We used a 3D full-wave field analysis to evaluate the optical properties and to optimize the appropriate size of spherical-shaped gold nanoparticles (Au NPs). We found a change in Au NPs’ radius from 5 nm to 50 nm, and an increase in spectral resonance peak depicted as a red-shift from 520 nm to 552 nm. Using this fact, important parameters that can be attributed to the LSPR sensor performance, namely the molecular sensitivity, FWHM, $\eta_{RIS}$, and figure of merit (FoM), were evaluated. Moreover, computational simulations were used to assess the optimized size (radius = 30 nm) of Au NPs with high FoM (2.3) and sharp FWHM (44 nm). On the evaluation of the platform as a label-free molecular sensor, Campbell’s model was performed, indicating an effective peak shift in the adsorption of the dielectric layer around the Au NP surface. For practical realization, we present an LSPR sensor platform for the identification of dengue NS1 antigens. The results present the system’s ability to identify dengue NS1 antigen concentrations with the limit of quantification measured to be 0.07 μg/mL (1.50 nM), evidence that the optimization approach used for the solution-based LSPR sensor provides a new paradigm for engineering immunosensor platforms.

## 1. Introduction

The novel optical characteristics of noble metal nanoscale structures expound their potential for medical applications and insights into new technology horizons. Light interactions with metallic nanostructures can prompt a phenomenon well known as localized surface plasmon resonance (LSPR), which is a response of coherent collective oscillations of free electrons around the nanoparticle (NP) [1,2]. Due to the LSPR phenomenon, plasmonic sub-wavelength structures are known to induce a robust localized field enhancement in their vicinity [3]. Another aspect of LSPR is to offer a plasmon spectral peak shift, which is generated due to a change in the shape, size and material composition of nanoparticles. Similarly, a clear spectral peak shift can be observed due to the change in the surrounding medium around the NPs [2]. Thereby, metallic NPs have been extensively studied for use in biosensing [4], photothermal therapy [5], surface-enhanced Raman scattering (SERS) [6], solar energy harvesting [7] and photodynamic therapy [8,9].

The LSPR-based optical sensor was explored as a low-cost, fast and reliable tool for the label-free detection of immunoreactions [2]. Recently, the reported LSPR biosensors were designed based on the electric field enhancement next to the nanoparticle [10]. Within the context of this fact, there is a dearth of research considering the NPs’ dimensions and the sensing performance of the LSPR device. A significant functional principle employed in LSPR biological sensing is reliant on the dependence of resonance spectral shift due to environment medium, size and shape [2]. In particular, such a plasmonic shift in the LSPR spectrum can be prompted by the direct adsorption of molecules to the surface of the metallic NP or by the specific binding of analyte molecules to NPs through functionalization with molecular recognition sites or ligands. Various examples of metallic NPs have been allowed to design biosensors for the detection of Alzheimer’s disease [11], proteins [12], antibodies [13], glucose-based sensing [14] and amyloid-beta oligomer detection [15]. Moreover, several important biomolecules, such as enzymes, DNA and proteins, have been associated with metallic nanostructures, providing a diversity of LSPR platforms for biomedical diagnosis [2,16].

In particular, only a few examples of LSPR sensors addressing neglected tropical diseases, such as dengue infection, can be found in the literature [17,18]. Dengue virus is the leading arbovirus viral infection, which is transmitted by the *‘Aedes’ mosquitoes*. The efforts to detect and control this virus are industrious, time-consuming and a public health demand. Presently, several clinical tests are used for diagnostics and therapeutics of dengue infection in the laboratory, such as a serologic test ELISA [19], a serotype-specific Immunoglobulin G (IgG) and IgM [20], as well as point of care tests and reverse-transcription polymerase chain reactions [21]. The merits and demerits of these methods have been exclusively reported and discussed based on distinct features, such as the limit of detection, durability, simplicity, commercial availability, rate of detection, accuracy, sensitivity and possibility of use for clinical applications [21]. In order to resolve such challenges, introducing new high-performance diagnostic methods and devices can be developed to manipulate novel perspectives on clinical procedures. However, in order to determine the performance of LSPR nanosensors, two important parameters (i.e., refractive index sensitivity and figure of merit) are explored, leading to a plasmon resonance shift that is generated by tuning the refractive index (RI) of the surroundings. For example, LSPR-based sensing has been broadly examined in the response of RI changes in the surrounding medium, such as solvents [1]. Therefore, the refractive index sensitivity ($\eta_{RIS}$) and figure of merit (FoM) are described as [2]:

(1) $$\eta_{RIS}=\frac{\Delta \lambda }{\Delta n_{m}};FoM=\frac{\eta_{RIS}}{FWHM}$$

where $\Delta n_{m}$ is the change in RI of the surroundings, and Δλ is the LSPR spectral peak shift. Moreover, the LSPR molecular sensor performance is predicted with these two factors. Therefore, for optimal molecular sensing, either $\eta_{RIS}$ should be higher or the LSPR spectrum should have a narrow FWHM. To obtain such optimal conditions, it is necessary to explore the proper size of the nanostructure, feasible material composition, facile shape of NPs and appropriate synthesis methods.

The emergence of new and straightforward synthesis methods to fabricate noble metal nanostructures (such as Au and Ag) has opened new horizons to explore in sensing applications and made it simple to diagnose such infectious diseases in a more economical, sensitive and rapid way due to their specificity and selectivity to various pathogens. Gold nanoparticles (Au NPs), in particular, due to their high stability, low cytotoxicity, bio-compatibility and their surface functionality, have been attractive as diagnostic probes [21,22]. Because of these outstanding features, Au NPs are commonly used in food safety [23], heavy metal ion [24] identification, hazard gas detection [25], photodynamic therapy (PdT) [8] and as a contrast agent [26]. Specifically, the use of Au NPs is favored as a biosensor due to their cost-effective fabrication, ease of use, durability and label-free sensing [2,3,17]. Moreover, the response of Au NP-based sensors due to a clear change in LSPR spectrum or color makes it convenient to interpret the results. Therefore, several studies have been reported on Au NPs for biological sensing applications in colloids [2,27,28]. Consequently, solutions based on plasmonic NPs can present an effective, rapid, more facile, cost-effective and higher-sensitivity option, encouraging us to manipulate Au NP probes as a label-free sensor.

This article explores an engineering procedure to establish a novel label-free LSPR immunoassay platform, accomplishing two requirements: high FoM and using stable Au NPs obtained through straightforward synthetic methods. Based on experimental results and a finite element method (FEM) simulation approach, the analysis relies on assessing the spatial distribution of the electromagnetic field enhancement near Au particles. The refractive index (RI)-based sensitivity and FoM, a function of optimal particle size, are evaluated to optimize the structure for predicting molecular LSPR sensor performance. By exploiting the optimal structure of Au NPs, we achieve the identification of dengue NS1 antigen as low as ∼1.5 nM at the optimal size of Au NP (radius = 30 nm).

## 2. Materials and Methods

### 2.1. Computational Modeling

**Figure 1:**
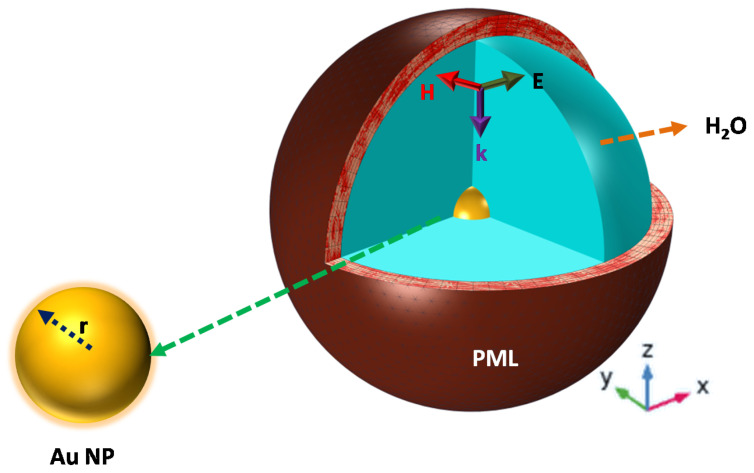
A 3D full-wave field analysis computational model with a PML domain and a spherical-shaped Au NP is immersed in surrounding medium (water).

A three-dimensional (3D) full-wave computational modeling is used to evaluate the optical properties of the NPs studied, exploring the finite element method (FEM) and based on the Radio Frequency (RF) Module of COMSOL multiphysics software. As represented in Figure 1, a single spherical Au NP is localized at the origin of a spherical computational domain, surrounded by a Perfect Matching Layer (PML), avoiding any kind of reflection artifacts. A homogeneous surrounding dielectric medium (H_2_O) has been assumed around the particle.

The time-harmonic E-field within the computational domain is given by [29]:

(2) $$\nabla \times \left(\frac{1}{\mu_{r}}\nabla \times E\right)-k{{}_{0}}^{2}\left(\epsilon {}_{r}-j\frac{\sigma }{\omega \epsilon_{0}}\right)E=0,$$

where *k_0_* is the free space wave-number, and σ and $\mu_{r}$ are the conductivity and permeability of the medium, respectively. The data for the dielectric function of gold nanoparticle simulations were obtained from the literature [30] and the incident electric field was set at 1 Vm^−1^. In addition, we employ the full-wave time-harmonic field theory, where the Au NP is illuminated with a uniform plane wave directed from the top PML surface towards the lower PML with the E-field polarized along the X-axis. To model an un-polarized light source for non-symmetric NPs, each simulation was performed twice, once with the incident E-field polarized along the X-axis and then with the E-field polarized along the Y-axis for NPs, and the results were averaged from these two calculations. However, for symmetrical NPs, un-polarized light is unable to affect the optical properties of the structure [31]. The model has been validated by comparison with Mie theory, as reported in [32].

The solutions of Maxwell’s equations were further manipulated to obtain scattering ($\sigma_{sca}$), absorption ($\sigma_{abs}$) and extinction ($\sigma_{ext}$) cross-sections. The optical cross-sections of the sub-wavelength particles are evaluated as [29]:

(3) $$\sigma_{sc}=\frac{1}{I_{0}}\int \int \left(n.S_{sc}\right)dS$$

(4) $$\sigma_{ab}=\frac{1}{I_{0}}\int \int \int QdV$$

(5) $$\sigma_{ext}=\sigma_{ab}+\sigma_{sc}$$

where n is the normal from the particle center. Further, *S_sc_* is the scattered-intensity vector over the surface area (*S*) of the simulation domain, *Q* is the heat loss density [29] and *V* is the particle volume.

In order to obtain computational analysis, the dielectric function of Au sub-wavelength structures related to exploring optical properties becomes a complex quantity under the influence of an external incident field, thus comprising a real part and imaginary part. The real part ($\epsilon_{r}$) and the imaginary part ($\epsilon_{i}$) of the dielectric function indicate a free-electron or Drude model [33], which can be expressed as:

(6) $$\epsilon_{\omega }=\epsilon_{int}\left(\omega \right)+\frac{\omega_{p}^{2}}{\omega (\omega +i\gamma )}$$

where γ presents a phenomenological scattering parameter, $\omega_{p}$ is a plasmon frequency and $\epsilon_{int}$ refers to the inter-band transitions. By analyzing the Drude model, the contribution of the γ parameter depends on the intrinsic properties of the material and from interface scattering by sub-wavelength NPs. The equation includes the scattering parameter given as $\gamma =\gamma_{bulk}+\gamma_{sca}$. As reported [30], the Au bulk damping constant ($\gamma_{bulk}$) and Fermi velocity ($V_{f}$) possess values such as $\gamma_{bulk}$ = 15 ${\left(fs\right)}^{-1}$ and $V_{f}$ = 1.4×10${}^{6}$ ms${}^{-1}$, respectively. The scattering parameter ($\gamma_{sca}$) is obtained as ($\gamma_{sca}$) =$\frac{AV_{f}}{L_{eff}}$, where $L_{eff}$ and *A* are the effective path length and scattering efficiency, respectively [5]. Moreover, it is clear that the dielectric function is frequency-dependent on the spontaneous polarization and the dielectric constant decreases as the NP size increases [34]. It is also a fact that reducing the $L_{eff}$ may tune both the real and imaginary parts of Au dielectric function. The increase in the imaginary value ($\epsilon_{i}$) causes the loss of NPs, while a small negative value of $\epsilon_{r}$ indicates lower polarizability of metallic NPs, as reported in [34].

### 2.2. Extinction Spectrum Setup

The optical spectrum is obtained in the visible and near-IR spectrum (400 to 1100 nm) using an Ocean Optics spectrophotometer (spectrum analyzer Ocean Optics HR +4000) with liquid samples embedded in quartz cuvettes of 1 cm width. A Halogen–Deuterium lamp was explored to illuminate the sample using fiber bundles with standard multimode fiber (62 μm/125 μm) to guide the light to the cuvette and collect the transmitted light to the spectrophotometer.

### 2.3. Preparation of Sensing Elements

The Au NPs of average radius 2.5 nm, 30 nm and 50 nm colloids were obtained as reported in our previous research [34]. Transmission Electron Microscopy (TEM) Analysis-MET ( Morgagni 268-D, FEI Company) was performed to characterize the NPs. The FEI Morgagni 268D is a 100 kV TEM equipped with an 11-megapixel Morada CCD camera. The TEM is employed for sample characterization. The particle size, shape and their modification were achieved through TEM high-resolution images.

**Figure 2:**
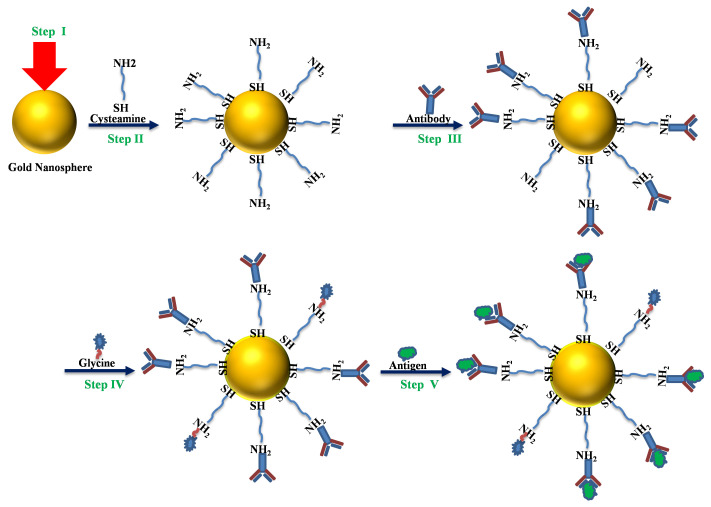
Schematic diagram of the label-free LSPR nanosensor: (step I) colloid Au NPs, (step II) ligand, (step III) anti-NS1 antibody, (step IV) glycine and (step V) adsorbed dengue NS1 antigen.

The cysteamine (Sigma Aldrich) thiol functional group (−SH) attached to the Au NP surface, while the amine functional group ($-{\mathrm{NH}}_{2}$) was bound with immunoglobulins IgG antibody [17]. The sensing platform comprised Au nanosphere colloid, mixed for an hour with cysteamine (2–aminoethanethiol) in ethanol. The ligand permitted immobilization with monoclonal anti-NS1 antibodies of the IgG class. Monoclonal anti-NS1 antibodies of immunoglobulins IgG class from rabbits (GenWayBio) were explored. To immobilize the anti-NS1 antibodies, the Au NPs and cysteamine (∼50 mM) were immersed for an hour in phosphate-buffered saline (PBS) solutions. On the platform development, the free-amine groups of cysteamine were blocked with ∼50 mM glycine solution (pH 6.5) to ensure that the antigens would bind only with anti-NS1 antibodies. To test if the developed platform could detect the presence of dengue NS1 antigen, a small amount of antigen solution was mixed with functionalized Au NP colloids for an hour. Different concentrations of dengue NS1 antigen solution from GenWayBio were prepared in PBS. All the steps for the sensing platform fabrication can be seen in the schematic diagram (Figure 2).

## 3. Results

### 3.1. Optical Properties of Au Nanoparticles

**Figure 3:**
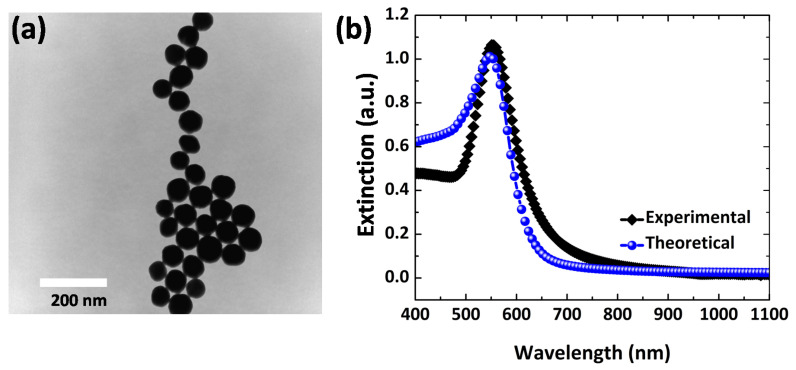
(**a**) TEM image of Au nanospheres and (**b**) extinction spectrum obtained both experimentally (black) and theoretically (blue). The average size of Au NPs for spectral and computational analysis is ∼50 nm.

Our analysis uses Au NPs with spherical shapes and uniform size distribution for dengue identification. As shown in Figure 3a, TEM microscopy reveals Au nanospheres uniformly distributed with a size of 100 nm and highly stabilized. On the other hand, the spectrum of the Au NP colloid shows a resonance peak at 550 nm, which is well matched by the extinction spectrum using the numerical calculation for particles with an average size of 100 nm. Figure 3b also confirms our FEM model’s accuracy, in great agreement with the experimental plasmon peak resonance (at 550 nm), encouraging us to further explore the optical properties for sensing device fabrication.

**Figure 4:**
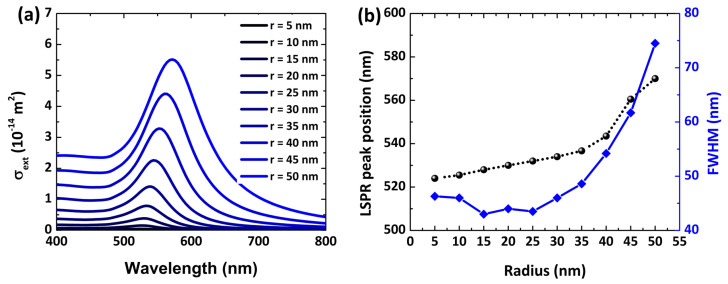
(**a**) LSPR spectral plasmonic resonance and (**b**) peak resonance wavelengths (black spheres) along with FWHM (blue cubes) as a function of particle radius. The radius parameter is tuned from 5 nm to 50 nm with a step of 5 nm, while keeping the surrounding medium constant (n = 1.33).

Further, by tuning the Au NPs’ size, the LSPR extinction spectra can be modulated over the visible to near NIR regime. Figure 4 presents the dependence of the LSPR extinction spectrum concerning the size of Au NPs. The plasmon resonance peak red-shifts on increasing the radius while keeping the surrounding medium unchanged. It is also clear that when increasing the radius of Au NPs, there is more interface scattering due to the larger effective path length, as reported in [35]. This interface scattering is responsible for LSPR spectral broadness, hindering larger nanostructures employed for label-free sensing [36].

The influence of interface scattering due to particle size variation is depicted in Figure 4b. The plasmon resonance peak shows a red-shift linearly as the particle radius tunes from 5 nm to 30 nm within the quasi-static limit. As the NP radius increases up to 30 nm, the interface scattering enhances, causing a broad FWHM of the LSPR spectrum. Consequently, increasing the FWHM reduces the FoM and affects the detection limit. Therefore, it is vital to explore the optimal size of NPs before the practical realization of sensing devices.

### 3.2. Localized Field Enhancement

**Figure 5:**
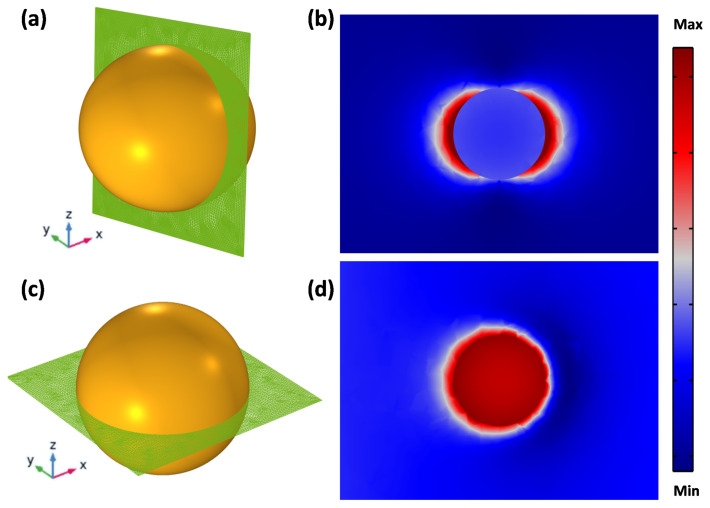
Cutting planes of individual Au NPs, parallel (**a**) and perpendicular (**c**) to the polarization field, and their respective E-field enhancement (**b**,**d**) distributions.

Due to light–particle interaction, LSPR has the ability to generate a strong localized field around the particle surface. Therefore, this localized field has been manipulated for several applications, such as SERS, fluorescent-based imaging and theranostic applications. In Figure 5, the near-field enhancement ($|\frac{E}{E_{0}}|$) color map of an individual structure is evaluated in different planes: the XZ plane parallel to the polarization field vector (Figure 5a,b) and the XY plane perpendicular to the polarization field (Figure 5c,d). As the polarization field is parallel to the cut plane, one can see the dipole lobes around the particle surface (Figure 5b), and these lobes disappear as the cut-plane is tuned perpendicularly to the polarization field (Figure 5d). Moreover, the E-field in the Au NP’s vicinity is robust and decreases as the distance grows from the surface in both planes.

**Figure 6:**
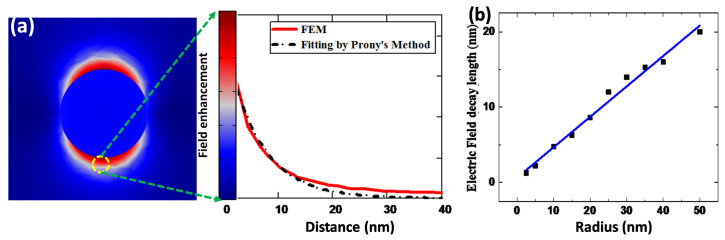
The calculation of $l_{d}$ values for gold nanosphere (r = 30 nm) using 3D full-wave field analysis in homogeneous medium n = 1.33 (**a**), and relation between particle radius versus $l_{d}$ (**b**).

Another important parameter for molecular LSPR sensing evaluation is the field decay length ($l_{d}$). For LSPR-based sensors, the $l_{d}$ values are <40 nm and, consequently, it depends on size, shape and composition [37]. Figure 6a depicts a 2D evaluation of the EM field distribution on the Au nanosphere surface. Figure 6b demonstrates the tendency of EM field decay length as a function of the radius of different-sized nanospheres. In order to obtain the field decay length, the field decay of a single NP can be fitted well by Prony’s method, evaluating the sum of real/complex exponential [38]. Moreover, Barbillon et al. procured sufficient behavior description of the EM field decay by using a single exponential fitting curve [39].

The field decay length grows linearly with the radius of the NPs, well fitted with linear regressions, with slope ∼0.19, and R^2^ = 0.997. For surface plasmon resonance (SPR) sensors, the $l_{d}$ values are of the order 200 nm to 300 nm [37], while, for LSPR sensors, the field decay values are a few nanometers. The lower $l_{d}$ values allow the exploration of lower concentrations of chemical or biological molecules.

### 3.3. Refractive Index-Based Sensing for Au NPs

**Figure 7:**
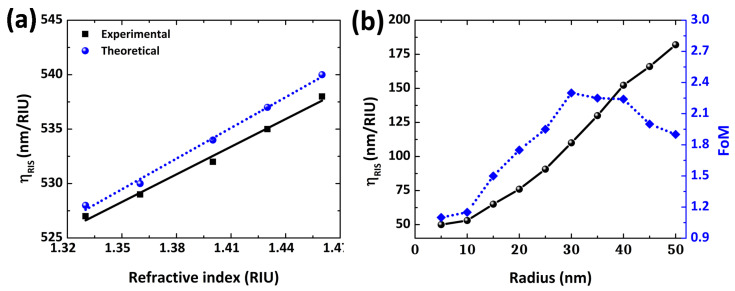
Evaluation of RI-based sensing on changing the surrounding medium (**a**) and $\eta_{RIS}$ with FoM as a function of NP radius (**b**).

Figure 7 demonstrates RI-based analyses of the LSPR spectral peak positions of Au NPs (r = 30 nm) in diverse surrounding media, such as water (n = 1.33), ethanol (n = 1.36), tetrahydrofuran (n = 1.40), dimethylformamide (n = 1.43) and polyethylene glycol (n = 1.46). As the RI of the surrounding medium increases, a clear red-shift of LSPR peak wavelength ($\lambda_{peak}$) can be observed. The LSPR peak position is linearly dependent on the RI of the surrounding medium. The nanoparticle size governs the refractive index sensitivity of an LSPR sensor. As the radius of the nanosphere increases, the particle surface to volume ratio changes, modifying the $\eta_{RIS}$ values. Figure 7b shows the effects of size on the RI sensitivity of the Au NP sensing platform. In Figure 7b, the radius of NPs is varied from 5 to 50 nm, leading to an increase in nanosensor $\eta_{RIS}$, from 50 to 172 nm/RIU. Furthermore, for sub-wavelength NPs (<30 nm), the light–NP interaction is mainly due to the absorption process. On the other hand, the optical scattering is governed by increasing the NP size beyond the quasi-static limit. It is reported that for the Au NP-based sensor, the $\eta_{RIS}$ is highly dependent on the NP size [31].

Regardless of the limitation of the quasi-static approach on small NPs, the calculated and measured values of LSPR peak wavelength and $\eta_{RIS}$ are in good agreement, as shown in Table 1. For instance, a 9% difference in the experimental and theoretical values of bulk sensitivity ($\eta_{RIS}$) was observed for a ∼30 nm radius of Au NPs. Only a 0.2% difference ($\lambda_{LSPR}$-error) is observed between the calculated and experimental LSPR peak wavelength values.

### 3.4. Molecular Sensing and Dengue Identification

As explored previously, the $\eta_{RIS}$ and $l_{d}$ values are dependent on Au NP size, such that the LSPR spectral peak shift of a plasmon sensor is conditioned to the nanosphere radius. The LSPR spectral peak shift (Δλ), due to the adhesion of molecules on the NPs surface, is expressed by Campbell’s model as [40]:

(7) $$\Delta \lambda =\eta_{RIS}\left(\eta_{ads}-\eta_{m}\right)\left(1-e^{-2d/l_{d}}\right)$$

where *d* represents the thickness of the adsorption layer, and $n_{ads}$ and $n_{m}$ depict the refractive index of the adsorbate and surrounding medium, respectively. Among the Au NPs of several sizes explored in our study, the Au NP with a radius of 30 nm shows the narrowest FWHM (Figure 4b), leading to a high FoM value to improve the detection limit.

**Figure 8:**
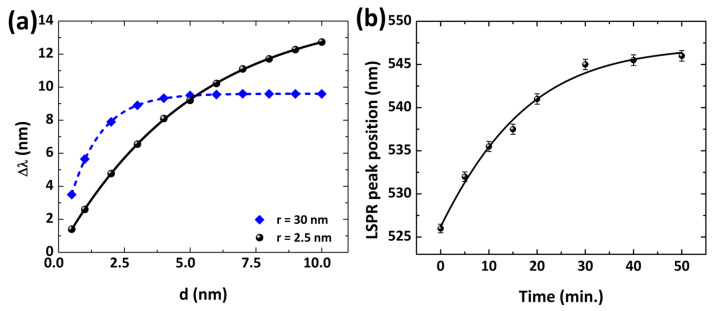
Plasmon resonance peak shift (Δλ) on increasing the dielectric shell around the particle (**a**) and the variations in LSPR peak positon due to cysteamine interaction with Au NPs as a function of time (**b**).

In our results, we examine, for molecular sensing, the adsorption of a molecular layer on the Au NP surface, generating a red-shift in the LSPR spectrum, which can be described by Campbell’s model. Figure 8 shows the LSPR spectral shift of the Au NP for the molecular sensing platform for 2.5 nm and ∼30 nm (radius), calculated using Campbell’s model (Equation (7)). As shown in Figure 8, we assumed that a single dielectric shell, with RI equal to 1.47 (∼cysteamine) and shell thickness (1 nm to 10 nm), covering the NP surface in water. By increasing the adsorbate layer thickness, the LSPR peak shift increases, for the 30 nm NP radius. This spectral shift is because of the change of *d* and the high $n_{ads}$ value. However, for a 2.5 nm radius of the Au NP, the LSPR spectral shift is not significant on growing the dielectric shell (>2 nm), as shown in Figure 8a. For particles with a radius greater than 30 nm, as the particle size increases, the RI sensitivity values are considerably enhanced (Figure 7b). However, an increase in NP size causes an enhancement of FWHM that ultimately reduces the FoM as well as the detection limit [34]. For instance, the Au NP of size 100 nm possesses an FWHM up to 70 nm. As a result, beyond the quasi-static limit for gold NPs, the LSPR sensor performance is highly affected due to interface scattering. Figure 8b presents the temporal evolution of the peak shift due to the plasmon resonance of Au NPs (∼r = 30 nm), with the adsorption layer of cysteamine (75 mM). It is a fact that cysteamine takes less than 30 min to bind 5 nm NPs [17] and a long time to achieve binding with particles larger than 50 nm NPs. Because of this fact, NPs with larger sizes (r > 25 nm) have a larger surface area and consequently require a longer time for the adsorption of cysteamine on their entire surface. Moreover, it could be found that when adding dense concentrations of cysteamine to the colloid containing Au NPs, there was no resonance peak shift observed, which caused the possible saturation of NPs.

In fact, the reason could be good RI sensitivity, FoM and sharp FWHM around the 30 nm radius Au NP for high plasmon peak shift. Using these features, we used ∼30 nm radius Au particles to detect the dengue NS1 antigen. The formation of amide bonds with a carboxyl group (−COOH) was observed based on the anti-NS1 antibody and amine group ($-NH_{2}$) of cysteamine.

**Figure 9:**
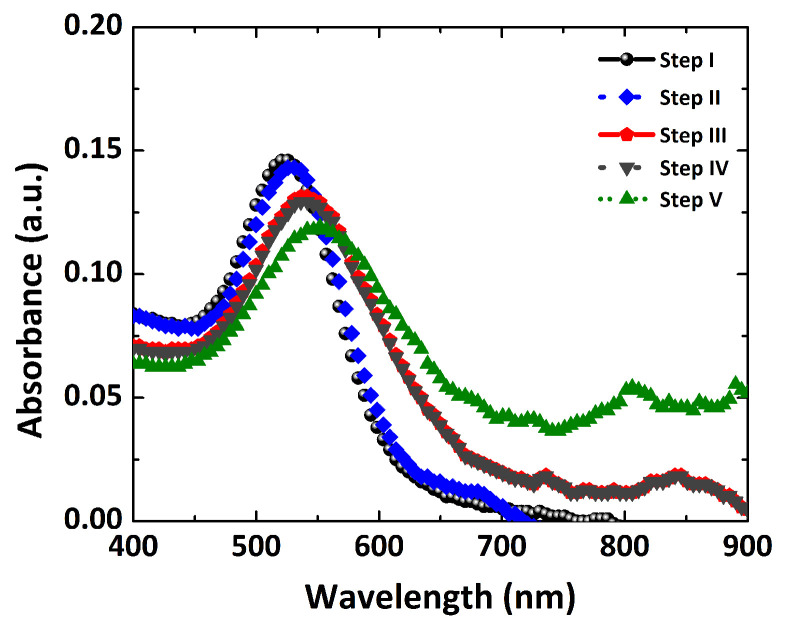
The label-free LSPR nanosensor in several steps: step I (Au NP colloid), step II (Au NP–cysteamine), step III (Au NP–cysteamine–IgG), step IV (Au NP–cysteamine–IgG–glycine) and step V (Au NP–cysteamine–IgG–glycine–dengue NS1 antigen).

For the anti-NS1 antibody, IgG was used in this label-free sensing platform. Moreover, an aqueous glycine solution was used to avoid the free binding of IgG antibodies with an amine group of cysteamine. The blocking can ensure the pure interaction of the NS1 antigen with the anti-NS1 antibody only. The dengue anti-NS1 antibody solution was prepared in buffered saline (PBS). Figure 9 shows the LSPR extinction spectrum of the Au NP platform, starting with a spectral peak at 526 nm. The adsorption of cysteamine on the Au NPs shows an LSPR peak shift (step II) 530 nm. The platform LSPR spectral peak, with the immobilization of IgG antibodies (1.0 µg/mL) and glycine (1.0 µg/mL), is shown with another LSPR spectral shift (step IV). One can see in Figure 9 that the gray spectrum possesses an LSPR spectral peak at 541 nm after immobilizing antibodies and glycine. After isolating the unbound cysteamine functional group (ligand) and immobilizing the anti-NS1 antibody, the LSPR biosensor is ready to detect the dengue NS1 antigen. Another significant LSPR spectral shift (step V) is observed when the dengue NS1 antigen is detected by the sensing platform (green curve in Figure 9). The obvious LSPR spectral peak shift could be examined at ∼12 nm after binding with an amide bond of the anti-NS1 antibody, as depicted in Figure 9 (step V). This spectral shift is reported to be higher after antibody–antigen binding in comparison with Camara et al. [17]. The LSPR extinction spectrum clearly exhibits a red-shift, as represented by the LSPR spectrum (green) at a very low concentration of 0.07 µg/mL (1.5 nM).

Theoretical results reveal that, for shells with a higher effective refractive index and longer thickness, using a ∼30 nm radius NP would lead to LSPR sensors with high sensitivity. However, the choice of the Au nanostructure with the best performances is, therefore, dependent on the RI value and adequate thickness of the adsorbate molecules, which might be measured by the use of two-medium or two-color approaches in classical SPR spectroscopy [41]. Table 2 summarizes our results with a recently published LSPR-based platform with and without a substrate.

## 4. Conclusions

We proposed an LSPR platform for biosensors based on 3D full-wave field analysis evaluation. The LSPR sensor was designed and fabricated based on the sensing performance using structure optimization by tuning the Au particle radius from 2.5 nm to 50 nm. We have obtained optimized performance with high FoM (2.3) and sharp FWHM (44 nm) at optimal NP size (r = 30 nm). For molecular identification, Campbell’s model presented a significant LSPR spectral shift due to the molecular adsorption layer on the Au NP surface. Furthermore, we applied an optimized Au NP-based sensing platform to functionalize monoclonal anti-dengue antibody with dengue NS1 antigen. Our results propose that the sensing ability of the solution-based platform is as low as 1.50 nM. Moreover, the optimized approach employed in this work can be extended to other nanomaterials, establishing a new paradigm for exploring a solution-based sensing platform.

## Figures and Tables

**Table 1:** Reported research on $\eta_{RIS}$ (nmRIU${}^{-1}$) and FoM using size-dependent nanostructures.

NP Size	Experimental Analysis		Thoeretical Analysis	
	$\eta_{\mathrm{RIS}}$	FoM	$\eta_{\mathrm{RIS}}$	FoM
2.5 nm	60	1.1	50	1.2
30.0 nm	84	2.0	83	2.3
50.0 nm	178	1.8	185	1.9

**Table 2:** Recently reported $\eta_{RIS}$ and dengue identification via label-free LSPR platform using Au NPs.

Nanostructure	Type of Platform	$\eta_{b}$ (nm/RIU)	Molecular Identification	Reference
Au NPs	solution-based	110	1.5 nM	This work
Au NPs	substrate-based	89	1.2 μM	[4]
Au NPs	substrate-based	−	1.54 nM	[17]
Au NPs	solution-based	−	104 μM	[42]

## Data Availability

The data presented in this study is available on request from the first author.
